# Distinct configurations of protein complexes and biochemical pathways revealed by epistatic interaction network motifs

**DOI:** 10.1186/1752-0509-5-133

**Published:** 2011-08-22

**Authors:** Fergal Casey, Nevan Krogan, Denis C Shields, Gerard Cagney

**Affiliations:** 1 Complex and Adaptive Systems Laboratory, University College Dublin, Belfield, Dublin 4, Ireland; 2 Department of Cellular and Molecular Pharmacology, University of California, 1700 4th Street, Byers Hall 308D, Box 2530, San Francisco, CA, 94158, USA; 3 Clique Graph and Network Analysis Cluster, University College Dublin, Belfield, Dublin 4, Ireland; 4 School of Biomolecular & Biomedical Science, University College Dublin, Belfield, Dublin 4, Ireland

## Abstract

**Background:**

Gene and protein interactions are commonly represented as networks, with the genes or proteins comprising the nodes and the relationship between them as edges. Motifs, or small local configurations of edges and nodes that arise repeatedly, can be used to simplify the interpretation of networks.

**Results:**

We examined triplet motifs in a network of quantitative epistatic genetic relationships, and found a non-random distribution of particular motif classes. Individual motif classes were found to be associated with different functional properties, suggestive of an underlying biological significance. These associations were apparent not only for motif classes, but for individual positions within the motifs. As expected, NNN (all negative) motifs were strongly associated with previously reported genetic (i.e. synthetic lethal) interactions, while PPP (all positive) motifs were associated with protein complexes. The two other motif classes (NNP: a positive interaction spanned by two negative interactions, and NPP: a negative spanned by two positives) showed very distinct functional associations, with physical interactions dominating for the former but alternative enrichments, typical of biochemical pathways, dominating for the latter.

**Conclusion:**

We present a model showing how NNP motifs can be used to recognize supportive relationships between protein complexes, while NPP motifs often identify opposing or regulatory behaviour between a gene and an associated pathway. The ability to use motifs to point toward underlying biological organizational themes is likely to be increasingly important as more extensive epistasis mapping projects in higher organisms begin.

## Background

Since the 1990s, data sets have accumulated where large numbers of genes or proteins are associated with each other using a variety of experimental approaches [1]). Prominent examples of such data include physical protein interactions identified by the yeast two-hybrid [2,3] or affinity purification mass spectrometry [4,5] techniques, and genetic interactions where the non-multiplicative (e.g. synthetic sick or lethal) effects of disrupting two genes are reported [6-8]. Recently, a quantitative variant of the genetic interaction screen was developed, termed Epistasis-Mapping (E-MAP)[9], and to date several thousand interactions have been published. As well as information describing their interactions, many other types of information describing genes and proteins are available, for instance, where they are located in the cell, what types of biochemical function they carry out, and whether they are essential for viability or associated with impaired phenotype when they are disrupted (reviewed in [10].

A natural way of representing these types of data is using network graphs, with genes or proteins forming the nodes and the relationship between them describing the edge. The wide range of methods and approaches used for studying graph-based biological networks is reviewed by [11]. These graphs contain hundreds to thousands of nodes and even greater numbers of edges, and they are challenging for biologists to manually analyze, or even to visualize. Therefore, extracting biologically useful information from these networks is difficult. A number of interesting observations have been made based on the global topologies of biological networks. For example, it was noticed that many biological networks show scale-free topology, leading to proposals that this arrangement may contribute to network robustness, being resistant to removal of random nodes [12]. Other proteins however, are important for the integrity of scale-free networks and it has been proposed that these proteins (termed *hubs*) may have a corresponding importance for the cell. Another property observed in biological and other networks is *small-worldness*, meaning that all nodes tend to be connected to each other by a small number of intermediate nodes [13]. Methods for analysing networks constructed using both physical and genetic interactions have been reviewed [14].

Motifs or configurations that occur repeatedly in networks can be used to try and understand the underlying biology. Over- or under-represented configurations have been observed in a wide variety of networks, including biological networks such as protein interaction maps and ecological food chains [15]. In a pioneering example, Zhang and coworkers [16] searched an integrated *Saccharomyces cerevisiae *network, containing five data types (protein-protein interactions; transcriptional relationships obtained from chromatin immunoprecipitation (ChIP) studies; synthetic lethal interactions; correlated mRNA expression profiles; and sequence homology relationships) for 3- and 4-node motifs and found several classes of statistically enriched configuration. The frequency with which motifs are observed reflects the fact that complex systems often have a restricted number of favoured states among the vast landscape of possible states. Motifs have been classified into superfamilies, where different families of motif class are associated with different types of network feature in biological and other networks (e.g. the internet) [17]. For instance, motifs corresponding to temporal transcriptional control of metabolic enzyme expression in yeast were recently identified [18]. In addition, network motifs may play different roles in different contexts, for instance very similar motifs engaged in different functions have been described in single cell microbes and in nerve cells [15]. Other aspects of the relevance of network motifs to biology have been studied, including their role in the evolution of modularity [19], their relationship with network hubs [20], and their use in predicting physical protein-protein interactions [21].

While network motifs can be viewed as tools to facilitate understanding, they may also serve as functional units that are used again and again by nature to solve biological problems. In networks where flux information is available (i.e. quantitative directional information describing the rate of flow of chemical entities or information through a network), differential equations can be used to analyze the network and to find stable states. However, the majority of currently available datasets describing protein and gene interactions are static, describing merely the presence or absence of an interaction between the two genes in the network. In the case of E-MAPs a continuous score that may be positive or negative reflects the relative effect on growth rate of disrupting two genes [9]. Hou and coworkers recently introduced mixture modelling to generate a probabilistic E-MAP network to which they applied a Bayesian analysis to identify network modules [22], an approach that shows considerable promise. Here, we first asked whether enrichment for small triplet (3-node) motifs is present in E-MAP networks, and secondly whether enriched motifs are correlated with biological properties. Because it is likely that large-scale E-MAPs will soon be generated for mammalian and other model systems using RNAi technologies, our ultimate goal is to map the location of motifs within gene and protein networks relative to known biochemical pathways and protein complexes, in order to improve our understanding of how cells work.

## Results and Discussion

### Identifying triplet motifs in a compendium of yeast epistatic interactions

The strength of the epistatic effect between two genes in an E-MAP is expressed using an S-score, which quantifies deviation from the growth rate (approximated by measuring yeast colony size) expected if no epistatic interaction occurs between the genes [9]. Disruption of two genes can result in a phenotype where the growth rate is slower than expected (negative S-score), often corresponding to a situation where the product of one gene can compensate for the loss of the other. Alternatively, a phenotype where growth rate is faster than expected may arise (positive S-score), corresponding to a situation where the disruptive effect of loss of one gene is, in fact, reversed or relieved by loss of another [14,23,24].

**Figure 1:**
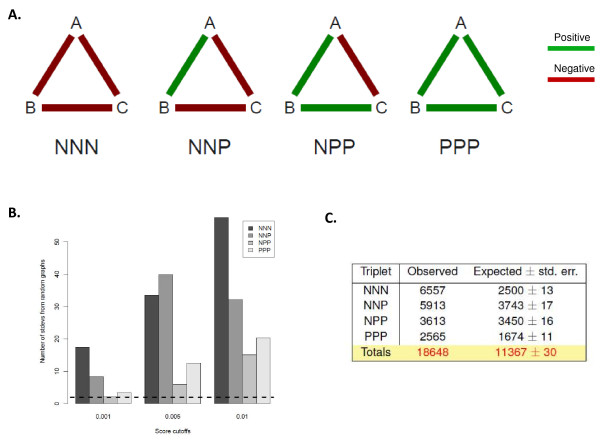
**Triplet motifs in epistatic interaction maps**. A) Positive (green) and negative (red) epistatic interactions can be used to link genes A, B, and C in four combinations. B) Counts of each triplet type were measured (using the number of standard deviations (SD) from the means obtained using random graphs) for datasets where epistatic interactions were defined as the gene pairs with the upper- and lowermost 0.1, 0.5 and 1 percentile S-scores. The dashed line represents an SD enrichment of 2.0, and corresponds to a p-value of ~ 0.05, assuming normality. C) The table shows the number of observed and expected counts of triplet motifs for the E-MAP compendium. This table was calculated using the 1-percentile definition of epistasis and expected values are based on random graph analysis.

We aimed to identify all the small triplet motifs composed of negative and positive E-MAP interactions (Figure 1A). We first assembled a compendium of epistatic interactions that included all published *S.cerevisiae *E-MAP interactions [9,25-27]. This dataset comprises a two-dimensional array of epistasis scores for pairwise knockouts, amounting to 2,237 yeast genes with 560,284 pairwise epistatic scores and 1,940,682 (about 78%) missing values. Genes that are tested in an E-MAP experiment are either complete knockouts (non-essential genes) or DAMP (decreased abundance by mRNA perturbation) alleles with reduced transcription (essential genes)[9].

In order to classify epistatic interactions as either positive, negative, or neutral (and to avoid bias introduced by arbitrary score cut-offs), we defined symmetric S-score cutoffs in the E-MAP data sets, with equal numbers of positive and negative interactions taken from the upper and lower *X *percentile. Here, *X *was specified by comparing the total number of each triplet type in the experimental E-MAP compendium with a randomised version of the compendium made by switching edges (the method of Milo et al. [17]). We chose the extreme 1% S-scores (i.e. most positive and most negative 1% of S-scores) for a cutoff that maximally enriches or depletes observed number of triplets compared to random graphs (Figure 1B). While studies to date in yeast suggest that negative interactions outnumber positive ones, it is unclear whether this reflects the nature of the yeast colony growth assay (which is more sensitive to negative interactions: a strongly negative interaction may have zero diameter/pixels whereas a strongly positive one will not have infinite diameter), or reflects a tendency in nature for negative interactions to dominate. Recent studies in cultured cells using alternative assays suggest a more even distribution [28], supporting the more conservative approach taken here. In order to reduce noise and to select only for genes that could be involved in a triplet, any gene that did not achieve two or more interaction scores beyond the threshold was excluded, leaving 1,752 genes (11,047 gene pairs). Hence, the set of binary interactions used to generate the triplets is not identical to the set of 1% highest and lowest S-scores in the original datasets; the set is available in Additional File 1. When arranged into triplets, these E-MAP compendium genes generated 18,648 triplet motifs (Additional File 2).

### Epistatically interacting genes pairs favour assembly into triplet motifs

Triplets are the simplest type of epistatic motif (apart from binary interactions) and are therefore a useful unit for studying the significance of motifs in E-MAP networks. A triplet motif contains a mutually interacting set of genes, and therefore is less likely to arise by chance, or through experimental noise, than binary interactions, or sets of three genes arranged in a linear manner. Additionally, higher order motifs have generally been found to be made up of combinations of lower order (doublet or triplet) motifs. We counted the frequency of triplet types (NNN, NNP, NPP, PPP; N and P indicating negative or positive epistasis) in the data using random expectation with standard error, finding that all types are enriched over random (Figure 1C). This confirms that genes interacting with two or more other genes prefer closed triplet motifs to linear or branched configurations, consistent with other reports showing that physical and genetic interactions tend to cluster together and to interact reciprocally within modules [29-31]. The distribution of triplet types at first appears to imply that negative interactions are more likely to be involved in triplets (NNN being more prevalent than PPP, NNP more prevalent than NPP)(Figure 1C). In fact, while there are slightly more negative than positive interactions in the triplets (55% are negative), the disproportionate count of NNN and NNP triplets reflects a tendency for a given negative edge to be involved in more triplets than a positive edge (average of 7.6 versus 5.6 for negative and positive pairs, respectively), thereby making the pool of unique negative edges produce relatively more negative-containing triplets. This arrangement also is reflected in the counts of expected numbers of triplets, since the graph randomization procedure we apply preserves the degree of each node, i.e. the number of positive and negative edges connected to the node.

The tendency to partake in triplet motifs suggests that epistatic interactions arrange themselves into structures reflecting higher levels of cellular organization, and that triplet motifs might capture some aspects of this structure that are not apparent from examining pairwise interactions in isolation. By their nature, the presence of negative interactions suggest an aligned or redundant function between two genes or their protein products (because the impairment of one is compensated for by the presence of the other), while the presence of a positive interaction suggests counterbalancing functions within a complex or pathway (because the impairment of one is masked or suppressed by the presence of the other). Interestingly, the distribution of positive and negative interactions per individual gene is often far from random. Even though the percentile cutoffs result in an equal number of positive and negative interactions being considered, we noticed that many genes interact predominantly through negative interactions or predominantly through positive interactions (Additional File 3). This type of polarity was previously noted by Segre and coworkers using computed models of epistatic interactions [32].

### Triplet members often share biological properties

**Figure 2:**
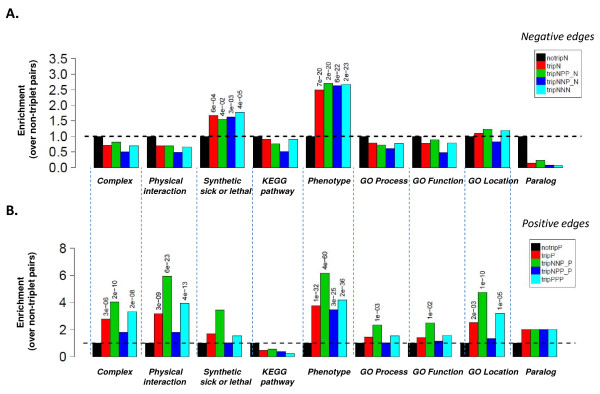
**Epistatically related genes pairs within triplet motifs show distinct patterns of shared biological properties**. A) Properties of negatively interacting gene pairs within triplets. B) Properties of positively interacting gene pairs within triplets. Enrichment for annotated properties is shown as fold-change relative to negatively (A) or positively (B) epistatically interacting pairs not in a triplet (Additional File 3). Fold changes greater than one that are statistically significant at p > 0.01 (Bonferroni corrected) are labelled with the p-value. Abbreviations: notripN = negative edge not in a triplet (black); tripN = negative edge in any triplet (red); tripNPP_N = negative edge in an NPP triplet (green); tripNNP_N = negative edge in an NNP triplet (dark blue); tripNNN = negative interaction in an NNN triplet (light blue).

If triplet motifs are biologically significant, one would expect that the members are involved in similar processes in the cell. In order to establish whether triplets are associated with previously described properties (e.g. involved in the same cellular processes and functions, physical or genetic interactions), we built a table of feature presence/absence per position in the triplet (rows) versus the triplet type (columns) (Additional File 1). We counted the number of biological properties shared among the nodes and edges of our set of E-MAP triplet motifs. The fold change in enrichment for negative interactions (Figure 2A) and for positive interactions (Figure 2B) show that many motif classes are enriched (or depleted) in individual network contexts. Overall, it is apparent that there is a general enrichment of biological features within triplets, as has been shown extensively for binary epistatic interactions [6,8,9]. For example, as expected, both negative and positive epistatic pairs within any type of triplet are several-fold more likely to share a phenotype. Negative interactions within triplets are enriched for shared genetic interactions, while positive interactions similarly are more likely to share a physical interaction or membership of the same protein complex. Interestingly, positive pairs within triplet motifs seem more likely than non-triplet pairs to share a cellular location, in contrast to negative pairs. Many pathways operate across cellular locations (e.g. signalling from cell membrane to nucleus during yeast mating), while most protein complexes are present in a single location at a given time, so this observations perhaps reflects the tendency for positive pairs to be associated with protein complexes or portions of biochemical pathways operating locally in the cell.

### Positive and negative edges within epistatic triplet motifs have distinct profiles of biological annotations

**Figure 3:**
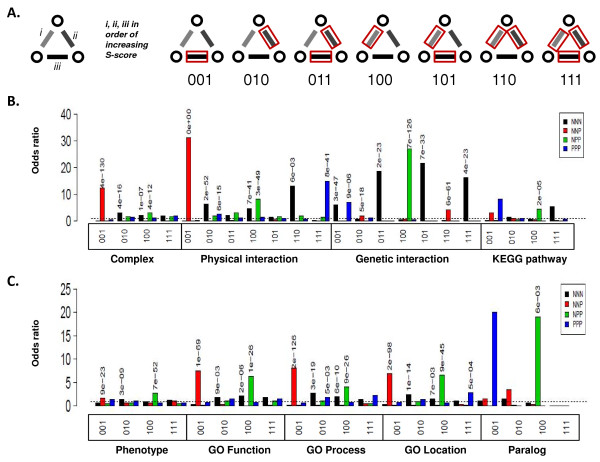
**Functional enrichment among individual genes pairs in epistatic triplet motifs**. A) Explanation of the scoring system. Edges in a triplet are arranged in order of increasing epistasis strength (i, ii, iii), with each position denoted '1' or '0' depending on whether the edge or adjoining nodes share a property. B) Odds ratios and associated p-values for protein complex membership, physical and genetic interactions, and shared biochemical pathway. C) Odds ratios and associated p-values for knockout phenotype, Gene Ontology Function, Process and Location, and presence of a paralog in *S.cerevisiae *(Additional File 3). In B and C, the dashed line indicates an odds ratio of one.

The distribution of triplet types among the compendium of E-MAP interactions reveals several distinct patterns of association with specific biological roles. In order to more fully understand the functional relevance of these patterns, we examined not only enrichment for biological annotations in the triplets themselves, but also the position *within *the triplet for any such enrichment (Figure 3A). In NNN and PPP triplets, the position of each epistatic pair appears equivalent, but the magnitude of epistatic effect can vary between them. Similarly, for NNP and NPP triplets, the association of a particular property (e.g. a physical interaction) with one pair rather than another may be informative. By focusing our analysis on the two main paradigms of protein organization in the cell, membership of protein complexes and membership of pathways, we can ask whether the occurrence of particular triplet motifs can be associated with particular arrangements of complexes or pathways. We therefore carried out Fisher exact tests on entries in a table that cross-tabulates features with triplet types (Additional File 4). This procedure yields an odds ratio and associated p-value for enrichment of biological features (Figure 3B, C).

A number of general observations can be made. a) Most striking is that the pair of positively interacting genes in an NNP triplet are highly enriched for physical protein-protein interactions and membership of protein complexes. Indeed Collins and coworkers [25] showed that genes encoding physically interacting proteins are more likely to interact positively than negatively. There is also enrichment for shared function, shared biochemical process and shared cellular location for the positive pair in an NNP triplet. b) The negative edges within NNN, NNP and NPP triplets often interact genetically, as expected. c) The negative edge of NPP triplets tend to share biological database annotations, location in the cell, to be members of the same biochemical pathway, and/or physically interact. They also tend to interact negatively with paralogs in the yeast genome. d) PPP triplets are enriched for physical interactions between all members of the triplet.

In order to confirm that these features are general properties of the yeast epistasis network, and not confined to a individual experimental method or laboratory, we repeated this analysis on a recently published dataset comprising over 5 million tested pairs [33]. The resulting enrichment patterns are almost identical between the two datasets (Additional File 5), suggesting that our observations are relevant at least for yeast epistatic interactions, and that these distinguishing properties may be useful for interpreting the potential roles of triplet genes in cellular biology. We next sought to analyze each triplet type, emphasizing the 'mixed' (NNP and NPP) triplets (because NNN and PPP triplets have been extensively discussed in the literature [22,26,29-31]).

### NNN and PPP triplet motifs

We extracted instances of NNN triplets where each edge is in a known genetic interaction, a combination that is highly enriched for this triplet class (p = 3 × 10^-6^), and plotted as a graph (Additional File 6). The triplets organize into structured (highly connected) modules, each containing genes involved in a related process. Overall, this NNN network confirms many earlier reports showing that networks of negative (i.e. synthetic sick or lethal) interactions are enriched for functionally related genes. For example, one cluster of negative interactions occurs between several DNA replication and repair enzymes, including members of the RAD52 epistasis group (RAD51, RAD52, RAD55), the MRX complex (RAD50), and the 9-1-1 clamp (RAD17), while another includes the proteasome genes PRE9, RPN4, and RPN10. Because genes interacting via negative epistatic interactions often carry out mutually supportive roles (the absence of one compensated for by the other), NNN motifs may be characteristic of genes acting cooperatively in functionally coherent roles.

Triplets of type PPP are strongly correlated with physical protein-protein interactions, and overlap extensively with known protein complexes (Additional File 7). By definition, the PPP-containing complexes exhibit positive internal (intra-complex) interactions. These contrast with the subunits of complexes such as the proteasome (mentioned above), whose genes tend to interact negatively. Notably Bandyopadhyay and co-workers [29] found that complexes containing predominantly negative epistatic interactions were more likely to contain essential genes than those with predominately positive interactions. Consistent with this, only two of the eight complexes depicted in the PPP sub-network (Additional File 7) contain essential subunits. Similarly, the finding that NNN-containing triplets tend to form an extended network, while PPP-enriched modules tend to be isolated, is consistent with an observations first described by Kelley and Ideker [34]. They showed that a combined genetic and physical interaction network in yeast could be better explained using a model where genetic interactions tended to separate physically interacting components ("between pathway") than one characterized by genetic interactions occurring within a physical complex ("within pathway").

### NPP and NNP triplet motifs

**Figure 4:**
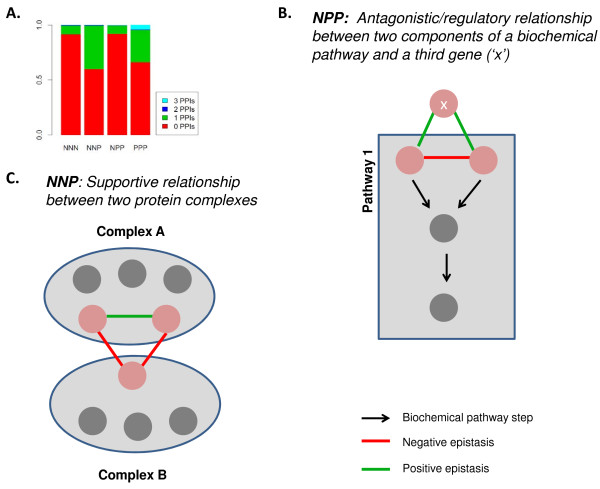
**Models for interpreting NNP and NPP configurations**. A) The fraction of epistatic triplet motifs containing zero, one, two or three edges where the gene products physically interact varies significantly between motif types. Arising from this and other observations, two models are proposed. B) NPP: Two proteins play partly redundant roles in a pathway, while they both interact with a third protein that acts in an opposing or antagonistic role, typical of a regulator. Two pathway examples, one forked and one linear, are shown. C) NNP: Different protein complexes coordinate or modify their activities by communicating with each other, mostly likely via a direct or indirect interaction between the proteins that span the two complexes.

Both NNP and NPP motifs are informative because they are strongly associated with configurations adjoining protein complexes and pathways. Because of the association between positive interactions and physical protein interactions, it might be expected that the higher the number of positive edges in a triplet, the more likely that one or more of the gene products take part in a physical interaction. In fact, while 8% of NNN motifs and 34% of PPP motifs have one or more physical interactions, the figure for NNP motifs is 40% (Figure 4A). In contrast, NPP motifs, despite containing two positive edges, are even less likely than NNN motifs to contain physically interacting gene products, only 7% having one or more physical interactions. As well as disfavouring the presence of physical interactions, the negative edge in NPP motifs typically contain gene pairs that share annotated biological properties, indicating that they take part in the same biochemical pathways (Figure 3B, C). NPP motifs therefore, may be common in situations where two genes play partly redundant roles in a pathway, while both interact with a third that may acts in an opposing or antagonistic role, typical of a regulator. A cartoon model showing possible scenarios for NPP motifs consistent with our observations is shown in Figure 4B and discussed below. By contrast, within NNP motifs, the positive edge is usually between a gene pair whose corresponding proteins co-purify in a protein complex (Figure 3B). Although the third gene in an NNP triplet (i.e. the gene spanned by two negative interactions) is usually not a member of the same protein complex as the other two, it is generally involved in another, different complex (shown by cartoon in Figure 4C). Further supporting this model, the components of NNP triplets show highly correlated epistatic interaction profiles within the triplet (Spearman correlation 0.79 ± 0.07 and 0.23 ± 0.08 for the positively and negatively interacting genes respectively), suggesting aligned function among the genes, and especially among the positively interacting genes. NNP triplets may therefore be associated with situations where different protein complexes coordinate or modify their activities by communicating with each other, mostly likely via a direct or indirect interaction between the proteins that span the two complexes (see below).

### NPP motifs often highlight an antagonistic or regulatory relationship between two components of a biochemical pathway and a third gene

The most common scenario for NPP motifs is that the negatively interacting gene pair is annotated within the same biochemical pathway but their gene products have not been shown to physically interact. In NPP motifs, the negatively interacting genes in turn interact through positive epistasis to a third gene, suggesting an opposing activity on the pathway. The third gene may regulate the pathway, because in its absence, over- or under-activity of the pathway increases cell growth. In order to test this model, we asked whether the third gene in NPP triplets displayed properties consistent with regulatory or substrate roles. Among the 456 genes in this category, those with annotations containing the terms 'regulator', 'activator' or 'repressor', suggestive or regulatory role, formed the majority of Gene Ontology terms enriched with a P-value > 10^-10 ^(Table 1). Consistent with potential regulatory roles, 113 genes occupying the 'positive' node of NPP triplets are transcription factors involved in the regulation of a wide variety of cellular pathways; for example BAS1 in purine and histidine biosynthesis, HIR2 in cell cycle regulated transcription of histone genes, OAF1 in peroxisome biogenesis, PHO2 in phosphate metabolism, RPN4 in proteasome degradation, STB5 in activation of multidrug resistance genes, and UME1 in meiosis (Additional File 4). Many of these proteins contain structural features prominent in regulatory activity such as the SANT, Zinc Finger, PHD, Myb, Homeobox domains (Additional File 4).

**Table 1:** Enrichement for Gene Ontology functional annotation terms among the positively interacting edge of NPP triplets

	Gene OntologyKeyword	%	P-value (Bonferroni corrected)	Fold Enrichment
*	regulation of transcription	11.40350877	8.836E-13	2.923169268

*	regulation of transposition	5.043859649	1.19207E-12	5.893402084

*	regulation of transposition, RNA-mediated	4.824561404	2.04113E-12	6.059954751

*	regulation of transposition	4.824561404	2.04113E-12	6.059954751

	membrane-enclosed lumen	27.19298246	2.7817E-12	1.784902043

	histone deacetylation	3.947368421	4.96909E-12	7.345399698

*	transcription regulator activity	16.44736842	7.24587E-12	2.225062309

*	regulation of biosynthetic process	12.28070175	7.64259E-12	2.64812693

*	regulation of macromolecule biosynthetic process	11.84210526	8.21877E-12	2.704442616

*	regulation of nucleobase, nucleoside, nucleotide and nucleic acid metabolic process	11.84210526	8.21877E-12	2.704442616

*	regulation of nitrogen compound metabolic process	11.84210526	8.21877E-12	2.704442616

	chromosomal part	14.47368421	1.10592E-11	2.380829016

*	regulation of macromolecule metabolic process	13.15789474	1.20581E-11	2.513634878

*	regulation of cellular biosynthetic process	12.06140351	1.82036E-11	2.623357035

	protein amino acid deacetylation	3.947368421	5.4495E-11	6.610859729

	response to DNA damage stimulus	13.59649123	9.22406E-11	2.355593696

* indicates annotations suggesting regulatory roles

**Figure 5:**
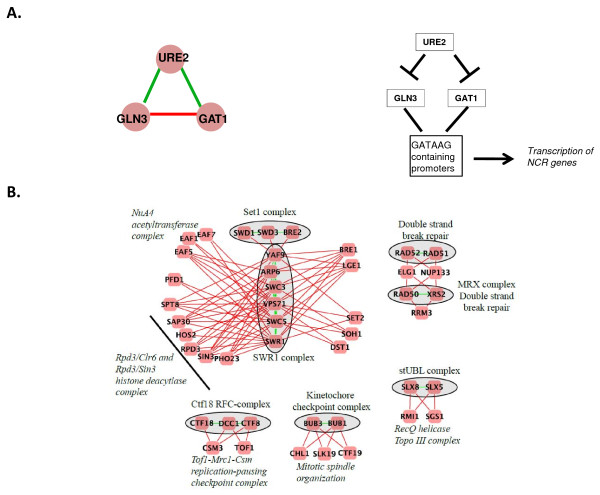
**Examples of NPP and NNP motifs in typical cellular contexts**. A) NPP motif where the 'positive' node, URE2, regulates GLN3 and GAT1 in the yeast nitrogen catabolite repression (NCR) pathway. B) A network made up of NNP motifs shows how the 'P' edge often separates two members of a protein complex, while the 'N' edges form a link to a different protein complex.

A good example of how NPP triplets can indicate such relationships is the URE2-GAT1-GLN3 triplet (Figure 5A). In this case, URE2 occupies the 'positive' (i.e. potentially regulatory) position, while the GATA transcription activators GAT1 and GLN3 are separated by a negative interaction. These genes are involved in the yeast cellular response to nitrogen availability [35]. GAT1 and GLN3 activate nitrogen utilization enzymes when nitrogen is limited, while URE2 inhibits this activity by confining GAT1 and GLN3 to the cytoplasm when nitrogen sources are readily available [36].

### NNP motifs often highlight a supportive relationship between two protein complexes

The presence of a physical interaction between the positively interacting genes of an NNP triplet is the strongest signal in the data set. Moreover, in our E-MAP compendium, the occurrence of NNP motifs is strongly associated with two protein complexes separated by a negative edge. This data supports a model where NNP motifs mark regions of the epistasis network where two different protein complexes coordinate their behaviour in a supportive manner [29,31]. Several such functional connections support this idea for the NNP network (Figure 5B). For instance six protein complexes involved in different aspects of chromatin biology are connected by NNP motifs (top left Figure 5B). Central to these interactions is the SWR1 complex, which is responsible for incorporation of the variant histone H2AZ [37]. The individual SWR1-C subunits are separated by positive edges, while interactions between SWR1 and the other complexes are negative. All these complexes play roles in gene expression regulation by catalyzing steps in the modification of chromatin. For example, the SET1 complex (also known as COMPASS) methylates histone 3 [38] while the NuA4 complex acetylates histone 4 [39]. Both these modifications are associated with active transcription, consistent with the idea that, since the intercomplex interactions are negative, the complexes may provide supportive or redundant roles during gene expression. Interestingly, two recent studies linking the function of the NuA4 complex to other chromatin modification complexes, including SWR1-C, highlight a coordinating role for EAF1 [40,41]. The Bub1p spindle checkpoint protein provides another example of NNP triplets spanning protein complexes with aligned roles (Figure 5B). Bub1p associates physically with the kinetochore via Skp1p, and it is proposed that this interaction is responsible for communicating a signal to the spindle checkpoint pathway that a kinetochore tension defect exists [42]. Thus, the BUB1-BUB3 positive interaction, reflecting the substrate-product relationship between these two proteins, is linked via NNP motifs to other kinetochore components, such as Ctf19p.

## Conclusion

In conclusion, we find evidence for a tendency of two triplet motif classes, NNP and NPP, to be associated with distinct modes of network arrangement in a large set of yeast epistatic interactions. In both cases, the motif members combine to fulfil general cellular roles in the cell, but NNP motifs favour supportive engagements between protein complexes, while NPP motifs tend rather to be associated with biochemical pathways where a third protein acts in an opposing, perhaps regulatory, role. These local network features are therefore potentially helpful for interpreting the biology underlying large epistatic networks, for which limited additional orthogonal data is available. The current yeast epistatic network is somewhat fragmented due to the nature of the E-MAP format (an individual E-MAP typically screens sets of 300-400 genes with a common biological theme). With increasing overlap between studies (i.e. linking different areas of cell biology), the use of motifs to infer biological organization is likely to become more powerful and more useful. Similarly, the use of RNAi technologies to carry out epistasis screens promises to extend the uses of motif analysis to higher organisms, including humans [43]. Our observations offer an initial approach to screening very large biological networks for motifs that highlight functionally relevant articulation points. This will facilitate the prioritization of further experiments to confirm hypotheses arising from the position of the motifs, as well as offering insights into how nature arranges epistatic relationships between genes in order to best advance the interests of the cell.

## Methods

### Biological data

The E-MAP interactions and S-scores were assembled from supplementary data of the relevant publications [9,25-27]. In cases where different S-scores were reported for the same interaction in different datasets, an average of scores was computed. Nine types of functional annotation or interaction data were used to correlate with the network motifs derived from the E-MAP data (downloaded 12 January 2010): Complexes ftp://ftpmips.gsf.de/yeast/catalogues/complexcat/; Protein-protein interactions http://www.thebiogrid.org; Genetic interactions http://www.yeastgenome.org; KEGG pathways http://www.genome.jp/kegg/download/; Phenotype http://www.yeastgenome.org; GO function/GO process /GO location http://www.yeastgenome.org; Homology (blastp on yeast protein sequences, scoring as homologs those gene pairs with and E-value cutoff of 0.01). The genetic interaction data set was processed by retaining only those entries in the interactions file that are denoted as 'Manual curation' and of type 'Synthetic Lethality' or 'Synthetic growth defect'. The ontology data were refined to increase the specificity of terms as outlined in Reference [16]. In order to assess correlation, for each gene in the E-MAP compendium that passed the score threshold, all terms are first mapped out to ancestor terms. Only those terms appearing between 2 and 40 times, maximizing both specificity and coverage, were retained. A similar ''fine-graining'' of phenotype terms was not possible due to the nature of the data, however we removed the general term 'viable'. Homology detection was performed by executing blastp on all yeast ORFs against themselves, and scoring as homologs those gene pairs with and E-value cutoff of 0.01.

### Graph randomization

The graph randomization algorithm for demonstrating enrichment of triplet motifs (Figure 1B and 1C) was that described in Reference [17]. Briefly, an edge switching approach is applied, whereby edges emanating from a given node are reconnected to other neighbor nodes in a manner that preserves the node degree (number of connections) of the original graph. In our implementation of this algorithm we ensure that the positive and negative degree of each node is preserved separately.

### Feature matching procedures

The feature matching procedures were chosen based on the graininess of the datasets. The presence of a matched feature was denoted with '1' and an absence by '0'. The individual epistatic interactions forming the triplet were ordered from most negative to most positive. For example, if the most positive (or least negative) edge in a triplet exhibited a feature while the other two edges did not, this would be denoted '001'. Features shared by gene pairs with the lowest E-MAP score (100), the middle score (010), or the highest score (001), were tabulated (Additional File 4). If any two edges in a triplet share a feature, then all three must also share that feature, an arrangement denoted (111).

## Abbreviations

E-MAP: Epistasis-MAP.

## Competing interests

The authors declare that they have no competing interests.

## Authors' contributions

FC, NK, DS and GC conceived and designed the experiments. FC and GC analyzed the data and wrote the paper. All authors have read and approved the manuscript.

## Supplementary Material

Additional file 1 **Table of features associated with members of triplet motifs**. Click here for file

Additional file 2 **List of triplet motifs extracted from the E-MAP compendium**. Click here for file

Additional file 3 **Genes showing most significant skewness towards positive or negative interactions**. Click here for file

Additional file 4 **Significantly enriched functional annotation terms associated with gene occupying the 'positive' node of an NPP triplet**. Click here for file

Additional file 5 **Functional enrichment among individual genes pairs in epistatic triplet motifs tested using an independent dataset**. A) Explanation of the scoring system. Edges in a triplet are arranged in order of increasing epistasis strength (i, ii, iii), with each position denoted '1' or '0' depending on whether the edge or adjoining nodes share a property. B) Odds ratios and associated p-values for protein complex membership, physical and genetic interactions, and shared biochemical pathway. C) Odds ratios and associated p-values for knockout phenotype, Gene Ontology Function, Process and Location, and presence of a paralog in S.cerevisiae. In B and C, the dashed line indicates an odds ratio of one. These data are calculated using data from Reference [33]. Click here for file

Additional file 6 **Network of selected NNN motifs with genetic interactions between the nodes**. Functional complexes or pathways are highlighted in elliptical bubbles. Click here for file

Additional file 7 **Modules of selected PPP motifs with physical interactions between the nodes**. Click here for file
