# Supplementary figures and images for: Distinct configurations of protein complexes and biochemical pathways revealed by epistatic interaction network motifs
Source: BMC Syst Biol. 2011 Aug 22;5:133. doi: 10.1186/1752-0509-5-133 (PMC3176491; doi:10.1186/1752-0509-5-133)

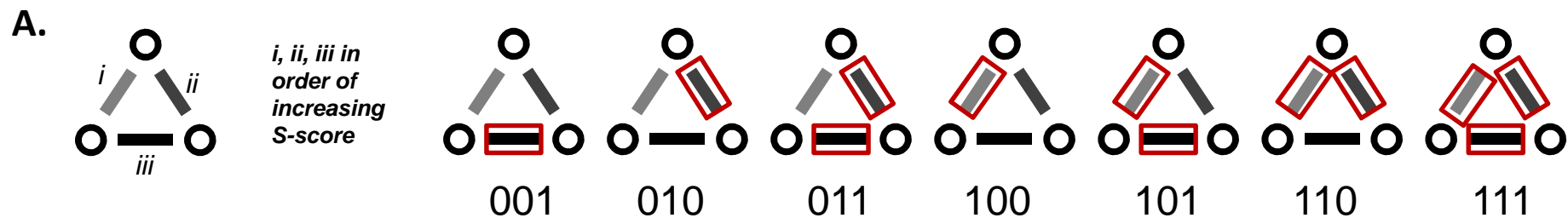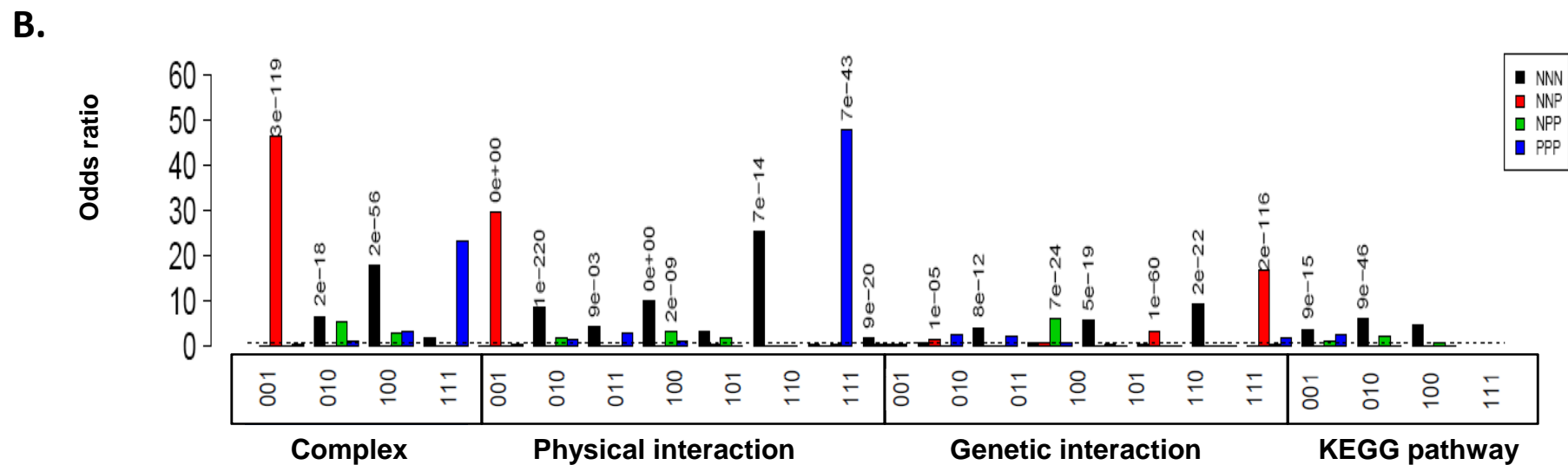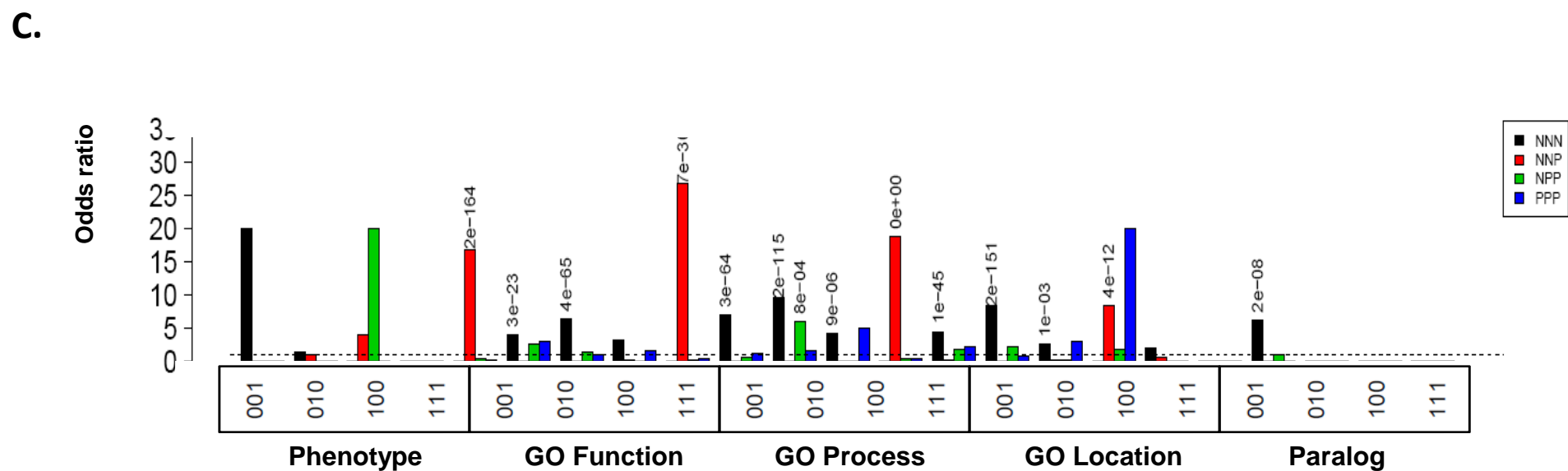

Supplement: Additional file 5 — Functional enrichment among individual genes pairs in epistatic triplet motifs tested using an independent dataset. A) Explanation of the scoring system. Edges in a triplet are arranged in order of increasing epistasis strength (i, ii, iii), with each position denoted '1' or '0' depending on whether the edge or adjoining nodes share a property. B) Odds ratios and associated p-values for protein complex membership, physical and genetic interactions, and shared biochemical pathway. C) Odds ratios and associated p-values for knockout phenotype, Gene Ontology Function, Process and Location, and presence of a paralog in S.cerevisiae. In B and C, the dashed line indicates an odds ratio of one. These data are calculated using data from Reference [33]. [file 1752-0509-5-133-S5.PDF]

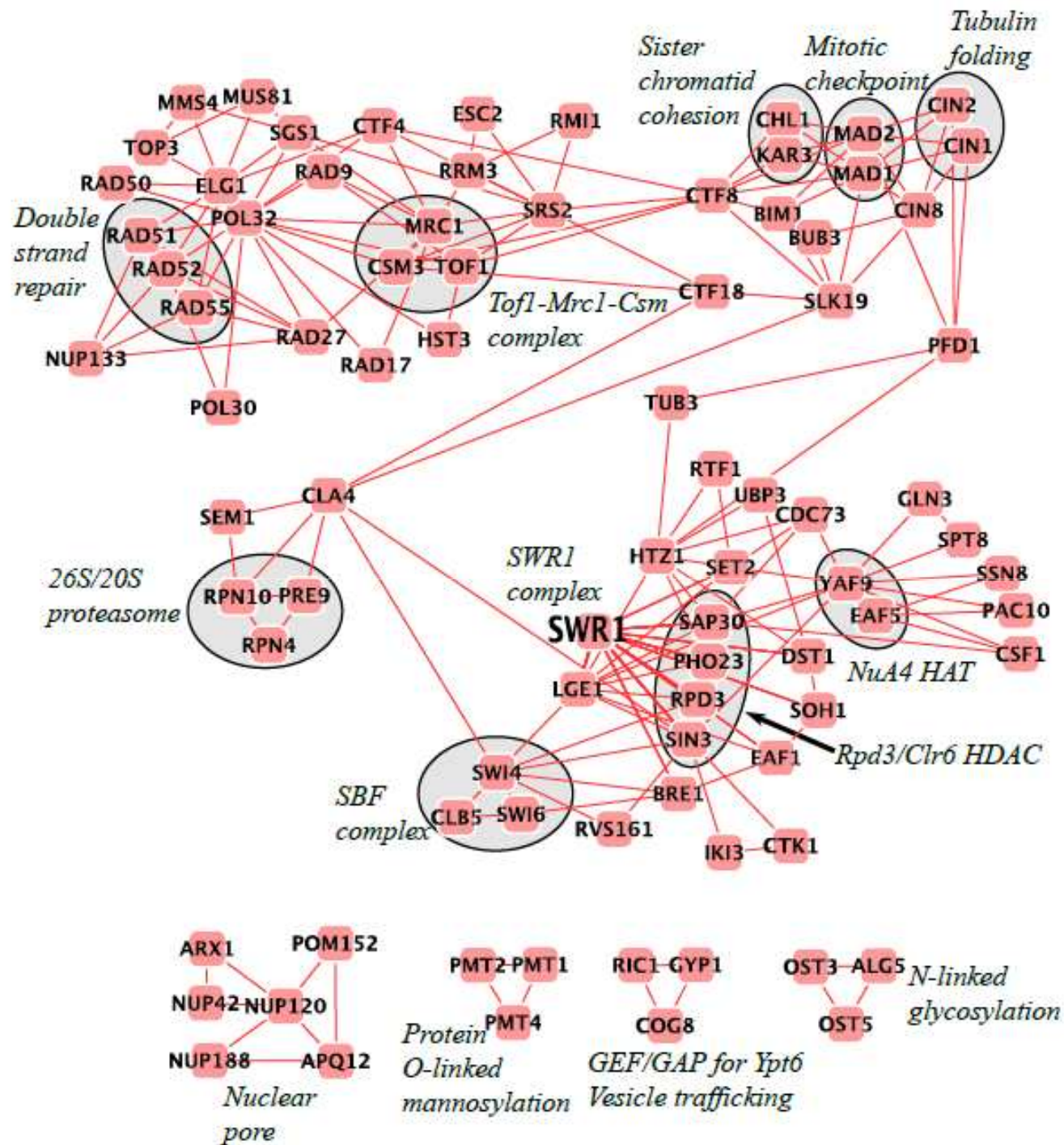

Supplement: Additional file 6 — Network of selected NNN motifs with genetic interactions between the nodes. Functional complexes or pathways are highlighted in elliptical bubbles. [file 1752-0509-5-133-S6.PDF]
