# Supplementary material for: Distinct configurations of protein complexes and biochemical pathways revealed by epistatic interaction network motifs
Source: BMC Syst Biol. 2011 Aug 22;5:133. doi: 10.1186/1752-0509-5-133 (PMC3176491; doi:10.1186/1752-0509-5-133)
Supplement: Additional file 7 — Modules of selected PPP motifs with physical interactions between the nodes. [file 1752-0509-5-133-S7.PDF]

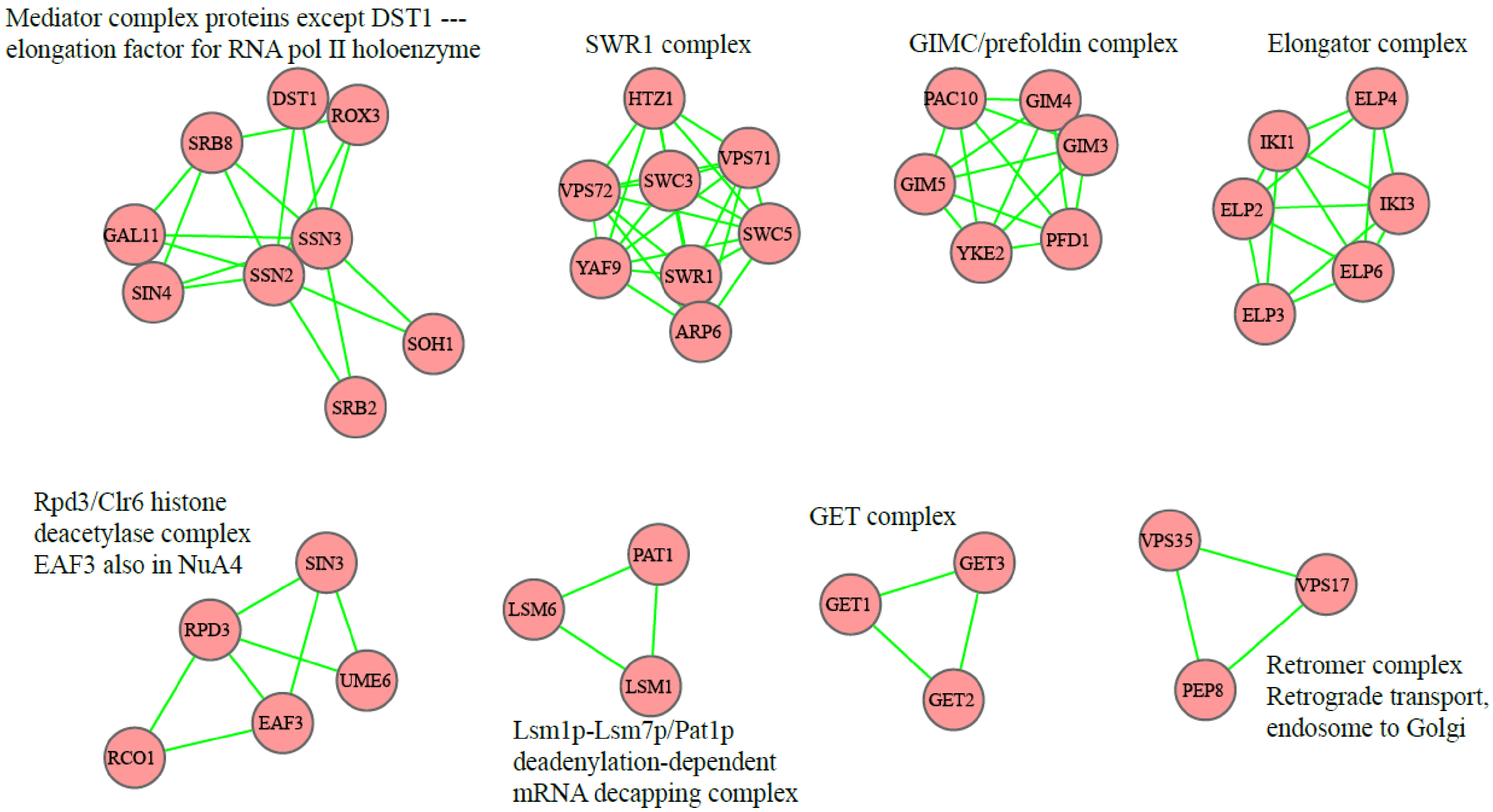
Mediator complex proteins except DST1 ---
elongation factor for RNA pol II holoenzyme
DST1
ROX3
SRB8
GAL11
SSN3
SSN2
SIN4
SOH1
SRB2
SWR1 complex
HTZ1
VPS71
VPS72
SWC3
SWC5
YAF9
SWR1
ARP6
GIMC/prefoldin complex
PAC10
GIM4
GIM3
GIM5
YKE2
PFD1
Elongator complex
ELP4
IKI1
ELP2
IKI3
ELP6
ELP3
Rpd3/Clr6 histone
deacetylase complex
EAF3 also in NuA4
SIN3
RPD3
UME6
EAF3
RCO1
PAT1
LSM6
LSM1
Lsm1p-Lsm7p/Pat1p
deadenylation-dependent
mRNA decapping complex
GET complex
GET3
GET1
GET2
VPS35
VPS17
PEP8
Retromer complex
Retrograde transport,
endosome to Golgi
